# Translating High-Throughput Phenotyping into Genetic Gain

**DOI:** 10.1016/j.tplants.2018.02.001

**Published:** 2018-05

**Authors:** José Luis Araus, Shawn C. Kefauver, Mainassara Zaman-Allah, Mike S. Olsen, Jill E. Cairns

**Affiliations:** 1 Unit of Plant Physiology, Faculty of Biology, University of Barcelona, Barcelona, Spain; 2 Global Maize Program, International Maize and Wheat Improvement Center (CIMMYT) Southern Africa Regional Office, Harare, Zimbabwe; 3 Global Maize Program, CIMMYT, Nairobi, Kenya

**Keywords:** field phenotyping, genetic gain, high-throughput, remote sensing

## Abstract

Inability to efficiently implement high-throughput field phenotyping is increasingly perceived as a key component that limits genetic gain in breeding programs. Field phenotyping must be integrated into a wider context than just choosing the correct selection traits, deployment tools, evaluation platforms, or basic data-management methods. Phenotyping means more than conducting such activities in a resource-efficient manner; it also requires appropriate trial management and spatial variability handling, definition of key constraining conditions prevalent in the target population of environments, and the development of more comprehensive data management, including crop modeling. This review will provide a wide perspective on how field phenotyping is best implemented. It will also outline how to bridge the gap between breeders and ‘phenotypers’ in an effective manner.

## Highlights

Although the development of effective field-based high-throughput phenotyping remains a bottleneck for future breeding advances, particularly those related to yield potential and stress resilience, progress in sensors, aeronautics, and high-performance computing is paving the way.

The growing interest in affordable solutions for remote-sensing approaches as well as data management may facilitate technological adoption by breeders.

However, phenotyping is more than just choosing the correct selection traits, deployment tools, evaluation platforms, or basic data-management methods.

Trial management and spatial variability handling, definition of the key environmental conditions that are prevalent in the targeted regions, and the development of more comprehensive data management, including crop modeling, are components of the phenotyping pipeline that need to be taken into consideration.

## Field Phenotyping Is a Bottleneck for Crop Genetic Improvement

Although there has been much success from the second half of the last century to now, the **genetic gains** (see Glossary) in yields of major crops such as wheat (*Triticum aestivum* L.) have stabilized or even stagnated in many regions of the world 1, 2, despite recent technical advances. This stagnation makes it more urgent to increase the efficiency of breeding. Limitations on phenotyping efficiency are increasingly perceived as a key constraint to genetic advance in breeding programs 3, 4, 5. Specifically, high-throughput **field phenotyping** may represent a bottleneck in conventional breeding, marker-assisted selection, or genomic selection, where phenotyping is a key informant for establishing the accuracy of statistical models [6]. Further, quality phenotyping is also required to evaluate the results of mutagenesis, genetically modified organisms [7], or even clustered regularly interspaced short palindromic repeats (CRISPR)/CRISPR-associated protein-9 nuclease (CRISPR/Cas9). This perception of phenotyping hindering genetic gain in major crops has aroused the interest of the scientific community into launching national, regional, and international initiatives [8]. The philosophy behind these initiatives is diverse and reflects the varied perceptions, priorities, or experiences of their promoters. In some cases, substantial emphasis is given to the development of top facilities amenable to phenotyping special traits; for example, root architecture and functionality under controlled conditions [5], or deploying ‘phenotyping field platforms’ that are permanently stationed in a given site 9, 10. However, while these facilities may help to advance our knowledge in the context of a research setting, the breeding community is still rather skeptical. Different factors have to be considered. The main concern for many breeders is that the controlled nature of many of the phenotyping platforms may not fully replicate the environmental variables influencing complex traits 3, 11, or adequately represent genotype by environment (G × E) interaction at the scale of large landscapes experiencing climate variability with large numbers of progenies being tested by breeding programs [12]. In fact, extensive phenotyping on a large (i.e., multitrial) scale via these platforms is perceived as something onerous [13] and the potential benefits may not justify the cost [3]. The vision of the private sector is also illustrative. In a recent presentation from Bayer CropScience (Dr Greta De Both) the perceived challenges that currently limit the adoption of new phenotyping packages for breeding are (i) validation of high-throughput field phenotyping; (ii) the need to develop flexible (mobile) and affordable approaches; (iii) the alignment of phenotyping under controlled conditions with targets for real (i.e., field) phenotyping; and, above all, (iv) data management, including user-friendly components and modeling and data integration. This review will provide insights on how these perceived challenges on genetic gain of crop plants may be addressed.

### Placing Phenotyping in a Wider Context

A fundamental concept in quantitative genetics and breeding is genetic gain. Genetic gain is the amount of increase in performance achieved per unit time through artificial selection [14] and can be defined as

[1] $$R_{t}=\frac{{ir\sigma }_{A}}{y}$$

where *R_t_* is genetic gain over time, *i* is selection intensity, *r* is selection accuracy, *σ_A_* is genetic variance, and *y* is years per cycle [15]. This equation provides the framework for measuring breeding progress. When placed in this context, to increase genetic gain, phenotyping can contribute toward improving selection intensity, selection accuracy, and even identifying new genetic variation. Obtaining precise and accurate estimates of genetic value (selection accuracy) is a fundamental goal of breeders and for that precision phenotyping is a fundamental issue [16]. Selection intensity is determined by the selection rate, that is, the proportion of the population selected from the total population [14]. Increasing the scale and cost efficiency of phenotyping can enable increased selection intensity. Larger population sizes allow greater selection intensity and improve the probability of identifying superior progenies [17]. Therefore, high-throughput methods are needed when phenotyping is implemented to efficiently screen larger populations. Plant breeding is *a priori* a costly process [18]. Furthermore, a critical aspect to the design of plant breeding programs is the allocation of limited resources between population size and replication [17]. The application of low-cost, high-throughput phenotyping tools to reduce costs will allow resources to be allocated to generation and management of larger populations, enabling an increase in selection intensity within a fixed budget. As selection pressure increases, genetic variability inevitably decreases. Phenotyping is crucial for ongoing efficient targeting of novel genetic variation to incorporate it into breeding programs for sustained long-term genetic gain [19].

**Figure 1:**
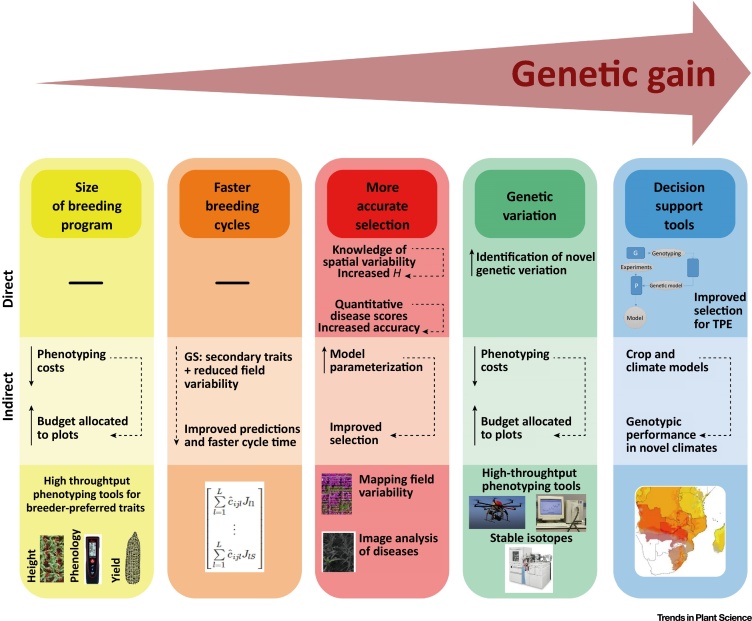
Key Figure: Five Pillars of Increasing Genetic Gain in Breeding Programs (Adapted from [20])

In summary, genetic gain within a breeding program can be accelerated in a number of ways [20], including (i) increasing the size of the breeding program to enable higher selection intensity, (ii) enhancing the accuracy of selection (higher repeatability), (iii) ensuring adequate genetic variation, (iv) accelerating the breeding cycles, and (v) improving decision support tools. In all of these components of the breeding pipeline, reliable high-throughput precision phenotyping is involved in a direct or indirect manner (Figure 1, Key Figure). Besides providing a global view of the phenotyping process, this review will focus on such factors and how they can be managed for the best outcomes when applied in the field. The final objective is to bridge the gap between breeders and the research community of ‘phenotypers’.

## Phenotyping Techniques

**Figure 2:**
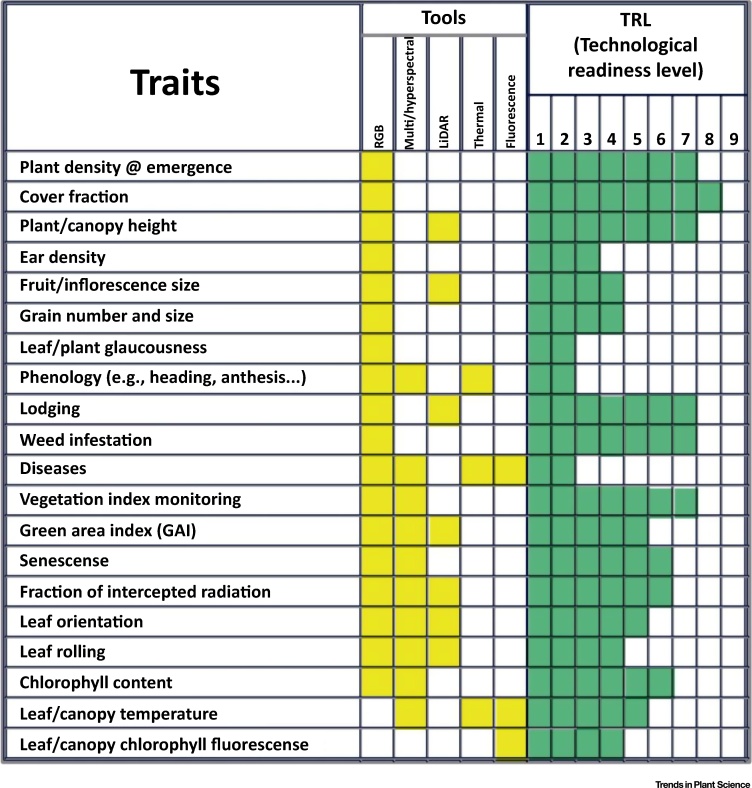
Summary of the Different Remote-Sensing Tools Most Commonly Used to Assess Shoot Characteristics of the Crop under Field Conditions, Together with a Comparative List of the Potential Applications and Their Level of Technological Development and Adoption. Different radar options are not included.

Much of what is currently considered high-throughput phenotyping is based on remote sensing (Figure 2). The most common types of remote-sensing devices used for crop phenotyping include multispectral, hyperspectral, fluorescence, and thermal sensors (particularly for ground-based phenotyping platforms), or imagers (which may be deployed from aerial platforms or at ground level, when several plots at a time are measured) using the radiation reflected or emitted by the canopy. Detailed information about the use of these devices for field phenotyping is extensive in the literature 3, 12, 21, 22, 23, 24. Furthermore, digital red–green–blue (RGB) cameras are widely used whatever the platform considered is (Box 1). In fact, most of the current low-cost approaches for crop phenotyping are based on exploitation of the possibilities opened by RGB images (Figure 2; see Figure I in Box 1).

**Figure I:**
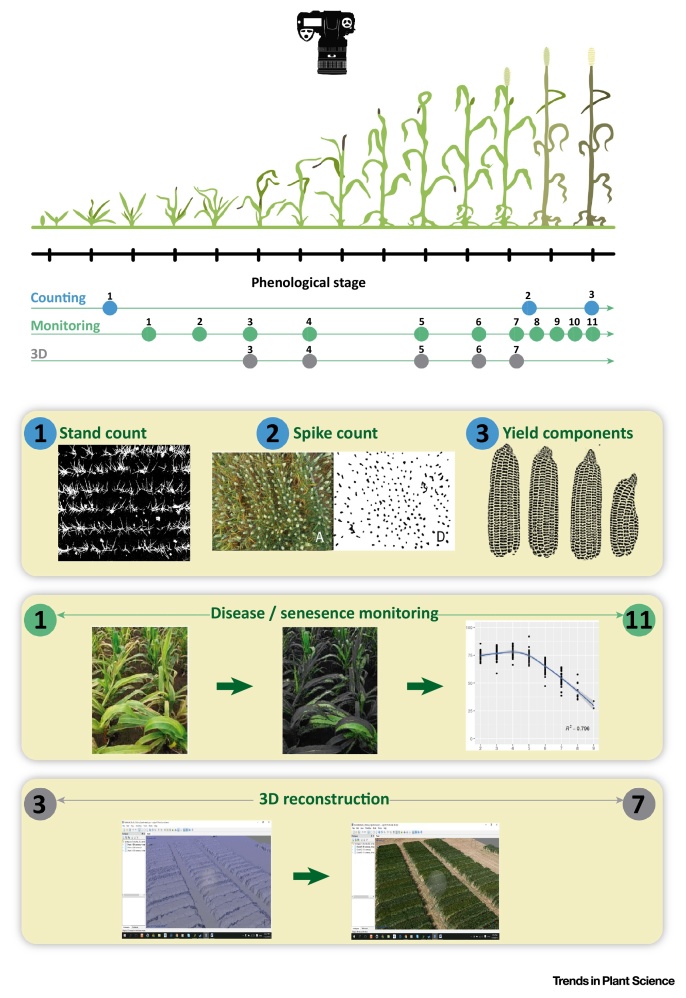
Examples of Potential Applications of Field Phenotyping with Red–Green–Blue (RGB) Images Produced by Conventional Digital Cameras. Different categories of traits are included: counting crop characteristics, monitoring plant/crop growth and development, and three-dimensional reconstructions. Part of the figure is redrawn from Fred Baret, INRA (EWG on Wheat Phenotyping to support Wheat Improvement).

Box 1 **The Use of RGB Images for Plant Phenotyping**

Vegetation indices derived from RGB images (Figure I) have shown their value as an affordable means of assessing genotypic differences in grain yield in response to a wide range of stress conditions including water stress [88], low nitrogen 67, 89, heat [90], or biotic stresses like yellow rust 91, 92. Interestingly, in all these examples, RGB indices outperformed spectral vegetation indices like the NDVI measured either at the ground level using a portable device with an active sensor or from an aerial platform using a multispectral camera.

In terms of sensors, the near future promises a widening of RGB image utilization. Several diverse factors support this prediction: (i) this is a really affordable way of phenotyping, given the low cost of high-resolution RGB cameras and the existence of open source software to formulate different categories of vegetation indices; (ii) the much higher resolution (at least four times) of RGB cameras compared with multispectral images makes the former more suitable for installation in aerial platforms; (iii) even from nanosatellites and microsatellites RGB images are amenable for important applications other than vegetation indices. To date, RGB imaging has proven its value not only in formulating vegetation indices that report on plant cover senescence, or the impact of foliar diseases, but also in a wide range of other applications (see Figure I in Box 3) in crop monitoring (phenology, disease detection), 3D reconstructions, and even counting (seedling emergence, ear density, other yield components). This includes very advanced areas like computer vision (Fernández-Gallego *et al.*, unpublished results). In the case of Miller *et al.* [52], the system for measuring maize ear, cob, and kernel attributes is being used by multiple research groups as an automated Web service running on community high-throughput computing and distributed data storage infrastructure.

Nanosatellites and microsatellites are emerging as an effective low-cost option for collecting data like sow date and yields on small farms across the developing world [93]. Further, the high resolution of RGB cameras may contribute to achieving images with pixels of 30-cm resolution, which is near the needs of many conventional phenotyping purposes.

Alt-text: Box 1

Remote-sensing tools allow assessment of physiological yield components that are clearly and conceptually related to crop productivity and stress adaption in terms of resource acquisition (radiation, water, nutrients, etc.), resource use efficiency, or downstream biomass partitioning 3, 21, 25. Changes in yield components impact yield potential as well as adaptation of the crop to unfavorable abiotic or biotic conditions.

However, equivocating remote sensing with high-throughput phenotyping is an oversimplification, because there are different categories of traits of an analytical nature, particularly grain/fruit quality traits, as well as analytical indicators such as stable (carbon) isotope, which have all proven their value 26, 27, 28, 29.

In the following sections, we highlight some remote-sensing techniques, which may be relevant in terms of special capacities and/or cost. In addition, light detection and ranging (LiDAR) and radio detection and ranging (radar), which use active sensors, will be also addressed (Box 2), because they are increasingly deployed to phenotype special features 3, 12, 21.

Box 2 **The Use of LiDAR and Radar for Plant Phenotyping**

Radar via synthetic aperture radar (SAR) systems is able to provide a wide range of plant physiological measurements relevant to high-throughput phenotyping in crop breeding: soil humidity, root characteristics, plant architecture, and counting fruits beneath the canopy [12]. Radar remote sensing uses the microwave portion of the electromagnetic spectrum, from a frequency of 0.3 to 300 GHz. Shorter wavelengths – for example, X-band imagery at 3 cm – are reflected from the top of the canopy, while longer wavelengths – for example, L-band imagery at 24 cm – normally go down to the ground and are reflected from there. The different frequencies of radar bands with their varying sensitivities allow detection of plant biomass and architectural details above ground (X and C bands) as well as other bands with varying depth range sensitivities in ground penetration radar with sensitivities to soil moisture and root systems underground (L and P bands) [94]. In addition, the polarization applied to each band also directly affects its sensitivity to various vegetation components. Different selections of frequencies and polarizations that alter SAR signals according to different plant physiological components may even make radar sensors, as still fairly portable active sensors, adaptable to fruit detection for automated fruit counting and even harvesting applications 95, 96.

LiDAR is another active sensor technique benefitting from technological advancements that have delivered more meaningful data at higher resolutions with less size and weight to the extent that it has become amenable to field plant phenotyping applications 18, 21, 97. In comparison to digital imaging and radar, LiDAR is an active, visible to near-infrared light sensing technique (mostly 800–1000 nm). It gives you enough signal at nonblinding power levels and a good mix of canopy penetration and return signal that is highly sensitive to plant canopy height and architecture; however, due to its use of shorter wavelengths, LiDAR is not capable of ground penetration. LiDAR processing techniques, such as high-density and full waveform, may provide different levels of detail on plant architecture, each offering in a sense, a trade-off between spatial resolution in horizontal (pixel size postprocessing) and vertical aspects (vertical differentiation in canopy architecture and biomass). In terms of applications in plant phenotyping, this may also apply to simple measurement of plant height at very high precision, including areas affected by lodging of crops.

Alt-text: Box 2

Remote-sensing tools are usually integrated into phenotyping platforms (Box 3). Some of these platforms, particularly stationary ones at ground level, and to some extent phenomobiles, are experimental facilities for developing new applications, or are special applications requiring customized solutions rather than being a generalized platform for wide-scale use in breeding. In addition, the increase in resolution capacity of sensors/imagers together with their miniaturization has lowered their cost. Improved resolution capacity and miniaturization coupled with increasing flight autonomy of unmanned aerial vehicles will contribute to further popularization of these categories of platforms in preference to the ground-based alternatives. Among the panoply of remote-sensing tools, those most frequently deployed in phenotyping platforms are RGB cameras, alongside multispectral and thermal sensors or imagers.

**Figure I:**
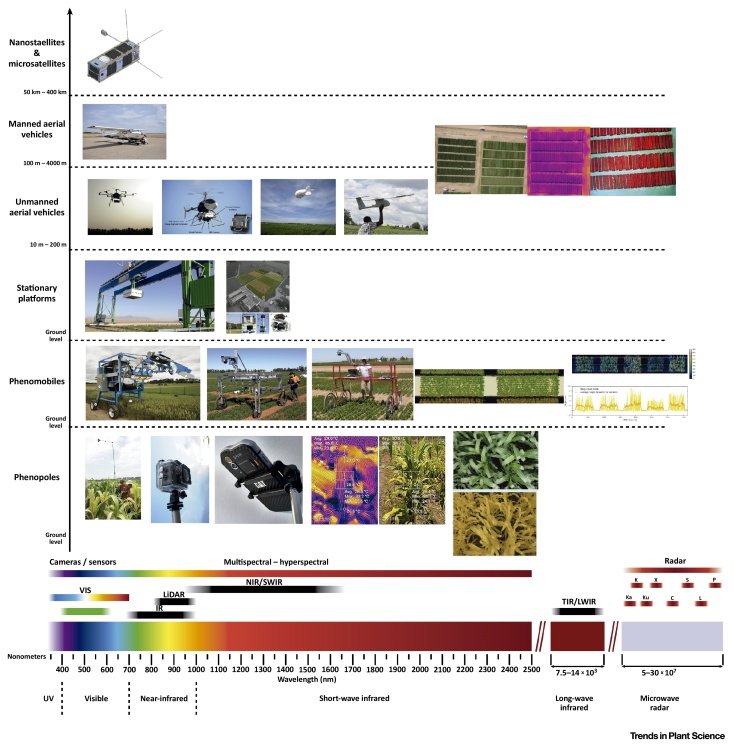
Different Categories of Potential and Actual Ground and Aerial Phenotyping Platforms, along with the Spectral Ranges Used for Different Remote-Sensing Tools. RGB cameras (VIS), multispectral and hyperspectral sensors and cameras, light detection and ranging (LiDAR) sensors, thermal sensors and cameras (TIR/LWIR), and the different categories of Radars. The horizontal axis is partially redrawn from [23]. The ground stationary platforms correspond to the Maricopa Agricultural Center (USA) and the ETH field phenotyping platform (Switzerland). The images of the phenomobile correspond to (from left to right) a proximal remote sensing buggy 21, 104, the phenomobile lite (https://www.youtube.com/watch?v=o8DmF7Y-GpE), and a phenocart [99]. The unmanned helicopter is from Chapman *et al.*[105]. IR, infrared; LWIR, long-wave infrared; NIR, near-infrared; SWIR, short-wave infrared; TIR, thermal infrared; VIS, visible.

Box 3 **Field Phenotyping Platforms**

The concept of the phenotypic platform is wide and ranges from platforms working under fully controlled conditions to field platforms or even platforms designed with a clear objective of ‘deep phenotyping’ of specific traits (e.g., grain quality traits), or elusive plant parts (e.g., roots). The choice of platform will depend on the scale of the intended work, including the sensors deployed, their resolution, and associated costs.

Within the category of field phenotyping platforms, the alternatives are still very diverse 18, 86 and encompass many levels (Figure I). These include at the ground level, ‘stationary’ or ‘fixed’ field solutions such as the cable-suspended multisensor system of the ETH Field Phenotyping Platform (Switzerland) [9], the Field Scanalyzer at Rothamsted Research, Harpenden (UK) [10], or the world’s currently largest robotic field scanner mounted at the Maricopa Agricultural Center (USA). At a smaller level, these include the different configurations of tractor-based systems, or the different categories (from phenomobiles, phenocarts) of specifically adapted carts, cranes, and linearly moving irrigation systems 18, 21, 98 to the use of ‘phenopoles’ and remote-controlled cameras. Within this category, smartphones [99] operated with a ‘phenopole’ may become even more of an alternative if they are linked to apps allowing data management and georeferencing. Moreover, smartphones have started to carry thermal cameras, either integrated or as add-ons, in addition to conventional RGB cameras. These smartphones are able to produce pictures of merged thermal plus RGB imaging, thermal temperature point measurements over RGB, and plain thermal camera modes.

Aerial platforms are a flexible alternative. Within this category and compared with the use of manned planes and even balloons, unmanned aerial platforms, also termed drones (including policopters, helicopters, and fixed-wing configurations), are attracting increasing attention due to their growing reliability and decreasing cost 3, 100, 101, along with their need of a smaller payload (with help to skip the increasing legal regulations about unmanned aerial vehicles).

In addition, over the coming years nanosatellites and microsatellites mounted with high-resolution RGB cameras may become an alternative to aerial phenotyping platforms 102, 103. Many nanosatellites and minisatellites are launched in sequence from the International Space Station, which is at 400-km altitude, and among which a good example may be the satellites by Planet Labs, with a submeter ground sample distance of 0.8 m (see https://www.planet.com/products/hi-res-monitoring/ and https://directory.eoportal.org/web/eoportal/satellite-missions/s/skysat).

Alt-text: Box 3

### Early Stress Detection

While the early detection of plant stress (detection prior to appearance of visual symptoms) remains a challenge for phenotyping [12], various techniques such as chlorophyll fluorescence, visible and infrared spectroscopy, and hyperspectral imaging have been tested 30, 31. Implementation of these methods under field conditions, particularly at the canopy level, remains a technological challenge. Recent advancements in the retrieval of sun-induced chlorophyll fluorescence due to improvements in sensors (hyperspectral imaging at a minimum of 1-nm wavelength range with incident light sensors 32, 33) as well as improved algorithms [34] allow advanced high-throughput phenotyping systems to measure these subtle early stress parameters at the canopy level in an agile manner [35]. As an alternative, the deployment of phenotyping sites with managed stress conditions (e.g., in the case of plant diseases, artificial inoculation or facilitated infestation) may allow use of the usual remote-sensing techniques.

### Multispectral Information: Beyond Formulating Indices

To date, most remote-sensing applications of multispectral/hyperspectral sensors and imagers have focused on using vegetative indices to infer overall plant status, with the normalized difference vegetation index (NDVI) being the most well-known option [18]. Although these indices generally can be informative, they use less than 1% of available spectra [12] and lack the ability to give detailed information on physiological processes [3].

Different studies have demonstrated that hyperspectral data (400–2500 nm) can be utilized to infer leaf chemical properties in various species 36, 37, 38. The hyperspectral reflectance approach to phenotyping is dramatically faster than traditional measurements, and offers a nondestructive method able to accurately assess physiological and biochemical trait responses to environmental conditions 39, 40. For example, naturally occurring Rubiscos with superior properties among the Triticeae tribe can be exploited to improve wheat photosynthesis and crop productivity [41]. In this context, hyperspectral sensors might allow *in situ* (i.e., field) fast evaluation of these traits (particularly using leaf adaptors). In addition, hyperspectral imaging has been tested for its ability to detect biotic stresses 31, 42. Moreover, the use of hyperspectral reflectance in the field as a high-throughput phenotyping tool to estimate complex traits like grain yield is promising 3, 43, 44, 45. Despite these promising applications, canopy spectral signatures are influenced by many external factors. For this reason, evaluation of single leaves using special adapters (with light source) may be required in certain applications to improve the overall trait assessment (e.g., [43]).

The category of hyperspectral assessments may also include near-infrared reflectance spectroscopy (NIRS), which is a basic tool in quality laboratories, for example, and NIRS imagers have been successfully mounted on harvesters to save time and sample handling costs. NIRS may be also deployed in the analysis of other traits with potential for phenotyping such as stable isotopes, and nitrogen and mineral content (see references in [3]).

## Phenotyping for Genetic Gain

As summarized in the previous section, the field of high-throughput phenotyping is rapidly evolving. There is a need to ensure that these advances find practical application in breeding programs and contribute toward increased genetic gain. While it is difficult to partition improvement within breeding programs to the adoption of specific technologies, placing technologies (including phenotyping) within the concept of genetic gain will assist in monitoring success. High-throughput phenotyping can contribute both directly and indirectly to genetic gain (Figure 1).

### Increasing Phenotyping Accuracy and Throughput

Obtaining accurate and inexpensive estimates of genetic value of individuals is central to breeding. Many routine traits such as stand establishment, phenology, abiotic stress severity, disease severity and progression, plant height, heading or flowering date, lodging, and yield components remain largely manual within breeding programs, particularly within the public sector 20, 46. Manual measurements are subjective, prone to human error, and lack robustness or repeatability. As a result, breeding teams may collect data on more replications or environments for some traits to improve trait heritability. Removing error and subjectivity from these measures at reduced cost would have direct savings for breeding programs. Moreover, visual scores, based on the naked eye, may not adequately capture the physiological status of plants. This was highlighted by a recent survey of genetic gain within the International Maize and Wheat Improvement Center’s (CIMMYT) Eastern and Southern Africa Maize Breeding program [20]. Substantially higher genetic gain for yield in artificially inoculated maize streak virus (MSV) trials compared with noninoculated trials was observed even though no change in visual scores for MSV were observed among hybrids tested from the 10-year interval, suggesting that many yield-impacting effects of MSV are not captured by the visual rating scale. Similarly, visual scores cannot distinguish between cosmetic (the persistence of greenness, which is not associated with extended photosynthesis) and functional stay-green [47]. While the accuracy of manual measurements of these traits is generally not documented, they are sometimes not reported due to low heritability or omitted due to problems measuring accurately across locations 20, 46. The development of high-throughput phenotyping tools to quantitatively measure key traits, particularly across locations, will increase selection accuracy at lower cost.

Recently, there have been many advances in the development of high-throughput phenotyping tools for ‘breeder-preferred’ traits (i.e., traits extensively used within breeding programs; Figure 2). Plant height sensors have been developed using a range of sensors including LiDAR, ultrasonic sensors, and RGB images 48, 49, 50. The maize private sector has moved toward quantifying ear traits using digital image analysis [e.g., https://www.google.com/patents/US9335313; https://www.google.com/patents/WO2017021285A1; https://www.google.com/patents/US20090046890]. New image analysis protocols are available to measure yield components using both a line scanner and conveyor belt and flatbed scanner 51, 52. The use of canopy temperature sensors combined with LiDAR to monitor green leaf area distribution has allowed the separation of functional from cosmetic stay-green [18]. Because of restrictions associated with phenotyping costs and time, dynamic traits are often measured just at several times points based on the best available knowledge of the most critical moments to measure a specific trait. The ability to take thousands of measurements per hour, combined with advances in high-density genotyping, has provided the unique ability to maximize genetic signal, improving the effectiveness of genomic prediction-based strategies 50, 53.

Many useful techniques remain either laboratory oriented 51, 52, are site specific [50], or require a pretreatment or calibration step as is the case for image analysis for morphological features of maize tassels, where tassels must be removed from the plant first prior to obtaining images in the field [54]. Therefore, there remains substantial scope for further technical improvement of techniques. Although effective high-throughput disease identification remains elusive, recent advancements in image processing (see [55] for a detailed review), namely, ‘deep’ and ‘machine’ learning (deep convolutional neural network, artificial neural networks, support vector machine, etc.) of big data, have demonstrated effectiveness for simultaneous crop pests and diseases identification 56, 57.

Manual measurements of key traits are time consuming and the application of high-throughput phenotyping tools could significantly reduce labor requirements. For example, the rate of manual measurements of plant height in rice (*Oryza sativa* L.) has been estimated at 45 plots per hour, compared with 3000 plots per hour using a phenomobile equipped with an ultrasonic canopy height sensor [50]. Similarly, estimating plant height through remote sensing in cotton required a small fraction of the time taken for manual measurements [58]. Substantial cost reductions using high-throughput phenotyping for routine traits with comparable selection accuracy would allow resources to be reallocated to strategies with potential to reduce cycle time (e.g., rapid generation advance or genomic prediction) or to increase population size, thereby increasing selection intensity. Although total cost reduction is a function of labor and time, the cost of equipment and training [58] may be a barrier to adoption of new phenotyping technologies. Initial training and phenotyping platform investment must be accounted for when considering cost–benefit value proposition of new phenotyping methods.

### Affordable Approaches

Adoption of recently developed high-throughput tools, carried out by ground and aerial phenotypic platforms, can involve large initial investment, particularly for use within testing networks covering large geographic areas. The challenge remains for high-throughput phenotyping to develop low-cost tools that can be applied across locations, especially when deployed in low- or middle-income countries where transportation may be expensive or difficult and where labor markets may result in relatively lower wages than in higher income countries.

While ‘robust sensors mounted on a field-deployable vehicle’ are considered imperative for a field-based high-throughput phenotyping platform [12], this is not necessarily dogmatic. In terms of tools and platforms, effective and expensive are not necessarily synonymous. There are a wide range of options for using RGB imaging to generate vegetation indices and other applications in crop monitoring (see Figure I in Box 1), together with flexible (e.g., ground hand-held and unmanned aerial) platforms (see Figure I in Box 3) that are easily deployable across a multitrial network. Some of these applications are also amenable for installation as apps on mobile phones 59, 60.

### Quality of Field Trials

Selection accuracy is a function of heritability, with increased repeatability increasing the selection response for the trait of interest. Genetic gains achieved during the last few decades through conventional breeding have been, in part, associated with an expansion of phenotyping networks 20, 61, 62. Expanding field networks across geographic locations can increase the problem of managing spatial variability (and G × E interaction) due to the increase in land area used, challenges with establishing new testing locations with no previous history, and the difficulty of covering large geographical distance [46]. Spatial variability inflates the estimated experimental error variance, reducing the ratio of phenotypic variance to total genetic variance. High residual error requires a greater number of trials for robust phenotypes 20, 46 and reduces accuracy of genomic predictions [63]. Detecting significant yield differences in genome-edited variants similarly requires a high signal-to-noise ratio [64]. A further challenge in collecting phenotyping data across field locations and years is varying environmental conditions [53], particularly for dynamic traits [12].

Within a field, many factors combine to generate microenvironments that differ from plot to plot, influencing yield and other traits [65]. When estimating genotypic effects it is important to correct for these factors. Compared with advances in phenotyping, fewer advances have been made toward increasing the heritability of measurements through either *a priori* or *a posteriori* control of spatial variability [66]. *A priori* methods of quantifying spatial variability are based on identifying existing trends within a field and can subsequently be accounted for using proper experimental designs. Vegetation indices, such as NDVI, have been successfully used for *a priori* mapping field variability [67]. Often correlated traits can be used as covariates during analysis, and some of these can be measured efficiently through remote-sensing techniques of phenotyping new tools. *A posteriori* control of the residual effects of using a model that provides a good fit to the data may become an alternative [66]. Spatial analysis, where global and local trends are fitted to the model, has played an important role in reducing spatial variability in conventional breeding [68] and improving prediction accuracies for genomic selection [69]. Open source models, using tensor product-penalized splines (P-splines), have improved the modeling of variability along the rows and columns of the field, thus increasing heritabilities 65, 70. High-throughput phenotyping has the potential to play an important role in the characterization of sets of environmental variables in training models that can be used subsequently to control these sources of data in postdata treatment.

### Crop Models and Decision Support Systems

While it is increasingly perceived that the data explosion associated with high-throughput phenotyping may hinder the full exploitation of such information, the use of simple summary statistics will also not suffice. Phenotypic data need to be acquired and processed, and analyzed rapidly in a quantitative and robust way into useful information that can be interpreted directly by end users. This involves the integration of quantitative genetics, statistics, and gene-to-phenotype knowledge, with genomic selection being a successful example of an empirical (i.e., statistically based) application 5, 61. It is notable that the final jump in technological readiness has come not from improved sensors or platforms, but rather from data-driven approaches and advancements in processing and data quantity using commercially available and affordable digital cameras 71, 72. Despite recent advances in data acquisition and processing, these large data sets have created new bottlenecks in data management [73], and advanced supervised algorithms require manual labeling of very large data sets for implementation [55], although they are potentially amenable to crowd sourcing development.

Crop models translate processes related to crop growth and development into mathematical equations [74]. This allows existing information from a limited number of field experiments to be leveraged and extrapolated to a wider range of conditions to predict and project how internal (e.g., trait or alleles) and external (e.g., climate or agronomic management) factors will influence crop performance 74, 75. In the agronomic context, crop models are being used in the assessment of climate change impacts and hotspots of climate change vulnerability, and in the prediction of performance across agro-ecologies and under novel climates 75, 76. Advances in genomics can enhance applications of modeling in breeding pipelines by elucidating the genetic basis of model-input parameters [61]. With genetic inputs, models can be used to simulate traits for a range of genotypes, locations, and years, demonstrating the combination of alleles required for specific environments [77]. High-throughput phenotyping platforms and tools offer a new avenue in the parametrization of models with genetic inputs [77].

However, mechanisms to capture the interaction of combined stress are often not properly fitted within models [78]. Moreover, modeling of biotic stresses has largely been through soft coupling of disease and crop models [74]. Therefore, predictions made by models can go beyond the assumptions and integrity of early models [79]. This is illustrated in the case of maize, where crop models were initially developed for high input, temperate cropping systems, with a significant gap in the predictive capability of models and decision support tools to accurately represent tropical soil dynamics, tropical germplasm, and low input production systems prevalent in the tropics 80, 81, 82.

Besides the aforementioned limitations, renewed interest in crop modeling has been associated with several international, multidisciplinary collaborations to update the ‘engines’ of existing models, including the Agricultural Model Intercomparison and Improvement Program (www.agmip.org), Global Future and Harvest Choice (www.harvestchoice.org), and the Climate Change, Agricultural and Food Security program (ccafs.cgiar.org). Significant progress has been recently made on ORYZA to improve the predictability of rice models under extreme conditions such as water- and nitrogen-stressed conditions [76]. In wheat, simulating temperature responses of physiological processes in 29 models accounted for more than half of the uncertainty in simulated grain yield [82]. Developing new temperature response functions in four wheat models reduced the error in grain yield simulations across seven global sites with different temperature regimes by over 40% [82].

## Concluding Remarks and Future Perspectives

Adoption of high-throughput phenotyping at a global scale will only be achieved by the end users (i.e., the breeders) if it is demonstrated as something valuable in terms of genetic gains achieved with resources invested. Moreover, in least developed countries, the public sector, rather than the private sector, continues to have a predominant role in crop breeding. To ensure that advances in phenotyping can be translated into yield gains in these countries, it is essential that low-cost phenotyping tools are developed. In that context, affordable, easy-to-handle, and reliable tools and platforms for large-scale (multitrial) field phenotyping may pave the way. Among such tools the wide range of applications for RGB images makes them good candidates.

To capitalize on advances in phenotyping and molecular technologies, greater progress is needed in areas of environmental characterization and data collection and management. Aligning breeding programs to future demands requires increasingly strategic choices: identification of rigorously prioritized, market-informed product profiles; understanding current and future target population of environments; balancing farmer and consumer needs; and accounting for value chain participant concerns. The engagement of breeding teams with climate scientists and crop modelers is perceived as necessary to address the challenges of climate change [83]. Fruitful areas of investigation include identifying key target breeding traits, the potential occurrence of multiple-stress factor interactions, the selection of trial sites, and the overall definition of appropriate methods for incorporating climate information into crop breeding programs.

Trait phenotyping and crop growth models are evolving to the point where breeders can access mechanistic information on the physiological determinants of plant adaptation for precise selection of **cultivars** suitable for the target environment [13]. At different levels, the use of big data will help to refine the geospatial targeting and requirements of new varieties [84], therefore addressing the genotyping × environment × management (G × E × M) interaction. Growth models fed with region-specific parameters including climate conditions, soils, and crop management will help to refine geospatial targeting, define regions, and therefore, accelerate breeding advancement [85]. This is especially the case when addressing future climate change scenarios and the expected increases in temperature [86]. Such an approach facilitates analysis of the genetic variation in plant performance for each environmental scenario. Indeed, a recent study in European maize has shown how feeding crop models with current and expected (climate change-driven) environmental variables from target environments and yield data from multilocation trials and their associated genetics has enabled detection of quantitative trait loci with specific roles for yield related to specific growing conditions [87]. It is anticipated that integrated utilization of this information can improve rates of genetic gain for important target environments [61], including environments expected to become more common under anticipated climate change scenarios (see also Outstanding Questions).

As the public sector breeding moves toward informal networks such as the proposed ‘European Consortium for Open Field Experimentation’ [8] and the Eastern and Southern African Maize Breeding Network 20, 46, the need to improve and standardize environmental collection is further reinforced. In that context, further contributions to improvements in the breeding effort will come from agreements among public research institutions and large phenotyping networks on common methodological standards for phenotyping protocols, data analysis, and information sharing.

Outstanding Questions Will genetic gain match the challenges imposed by the global (social and climate) change for the coming decades? Genetic gain is stagnated in many regions and the promises of a new ‘Green Revolution’ as the natural result of biotechnological advances have not yet been realized.

Even though high-throughput phenotyping is perceived as a bottleneck in breeding, it has yet to deliver. Is the dilemma of controlled versus field phenotyping in terms of yield or adaptation to abiotic stresses already solved considering that research and breeding application are not the same?

Will low-cost, high-throughput phenotyping tools be adopted regularly by breeders in the next decades? If so, are RGB cameras, mobile apps, and drones the natural candidates?

To what extent will the size of breeding programs need to be increased, considering improvements in accuracy of selection, ensuring adequate genetic variation, and acceleration of breeding cycles?

How will decision support systems and simulation models contribute to the breeding pipeline to predict future target environments in breeding, a component in molecular breeding, or design ideotypes?

## Glossary*

**Cultivar**: commercial variety.
**Field phenotyping**: phenotypic evaluation under the real (i.e., field) conditions experienced by the plant.
**Genetic gain**: amount of increase in performance achieved annually per unit time through artificial selection.
